# A Refined Crop Drought Monitoring Method Based on the Chinese GF-1 Wide Field View Data

**DOI:** 10.3390/s18041297

**Published:** 2018-04-23

**Authors:** Sheng Chang, Bingfang Wu, Nana Yan, Jianjun Zhu, Qi Wen, Feng Xu

**Affiliations:** 1 School of Geosciences and Info-Physics, Central South University, Changsha 410083, China; changsheng@radi.ac.cn (S.C.); zjj@csu.edu.cn (J.Z.); 2 Key Laboratory of Digital Earth Science, Institute of Remote Sensing and Digital Earth, Chinese Academy of Sciences, Olympic Village Science Park, W. Beichen Road, Beijing 100101, China; yannn@radi.ac.cn; 3 Key Laboratory of Metallogenic Prediction of Nonferrous Metals and Geological Environment Monitoring Ministry of Education, School of Geoscience and Infophysics, Central South University, Changsha 410083, China; 4 National Disaster Reduction Center of China, Beijing 100124, China; whistlewen@aliyun.com (Q.W.); xufeng@ndrcc.gov.cn (F.X.); 5 China Transport Telecommunications and Information Center, Beijing 100011, China

**Keywords:** crop drought, EVI2-based MPDI, GF-1 WFV data, relative soil water content, FVC

## Abstract

In this study, modified perpendicular drought index (MPDI) models based on the red-near infrared spectral space are established for the first time through the analysis of the spectral characteristics of GF-1 wide field view (WFV) data, with a high spatial resolution of 16 m and the highest frequency as high as once every 4 days. GF-1 data was from the Chinese-made, new-generation high-resolution GF-1 remote sensing satellites. Soil-type spatial data are introduced for simulating soil lines in different soil types for reducing errors of using same soil line. Multiple vegetation indices are employed to analyze the response to the MPDI models. Relative soil moisture content (RSMC) and precipitation data acquired at selected stations are used to optimize the drought models, and the best one is the Two-band enhanced vegetation index (EVI2)-based MPDI model. The crop area that was statistically significantly affected by drought from a local governmental department, and used for validation. High correlations and small differences in drought-affected crop area was detected between the field observation data from the local governmental department and the EVI2-based MPDI results. The percentage of bias is between −21.8% and 14.7% in five sub-areas, with an accuracy above 95% when evaluating the performance via the data for the whole study region. Generally the proposed EVI2-based MPDI for GF-1 WFV data has great potential for reliably monitoring crop drought at a relatively high frequency and spatial scale. Currently there is almost no drought model based on GF-1 data, a full exploitation of the advantages of GF-1 satellite data and further improvement of the capacity to observe ground surface objects can provide high temporal and spatial resolution data source for refined monitoring of crop droughts.

## 1. Introduction

Regional temperature and precipitation anomalies resulting from global climate change may cause droughts. Drought is a type of natural disaster that has significant impacts on the economy, society, food security and the human living environment [1]. Situated in the southeastern East Asian continent, China has a typical monsoon climate with uneven temporal and spatial distributions of precipitation as well as susceptibility to frequent droughts over large areas. The impacts on agricultural production are especially impressive. Zhou estimated the economic loss caused by drought at, up to 7.1–11.8 billion Renminbi (RMB) in the early 1990s [2]. During 2004–2007, each year drought affected approximately 16% of farmers nationwide, which resulting in an income loss of about 20% [3]. The nationwide drought happened from 2000 to 2001, the summer drought occurred in South China in 2003. Later in 2006, the once-in-a-century heavy drought taken place in Sichuan and Chongqing, and over large areas of North China in the winter and spring area suffered drought from 2008 to 2009 (an additional 0.112 billion mu of farmlands in China were affected by drought). The severe drought occurred in Southwest China in 2010, which caused nearly 20 billion RMB economic losses. From January to May 2011, five provinces in the middle and lower reaches of the Yangtze River suffered the most severe drought since the new China was founded. As China undergoes rapid economic development, the economic losses and the level of economic risk as a result of droughts increase significantly; this situation poses a severe threat to public security and national economic development. An urgent need to employ advanced technology exists to not only address the problem of real-time monitoring and detection of droughts over large areas but also improve the ability to rapidly respond to developing droughts and their changes. Chinese high resolution satellites has great potential to apply for disaster monitoring, damage assessment and related disaster risk management aspects [4]. GF-1 is the first satellite in of the Chinese High Resolution Earth Observation System program, which includes 7 different types of high resolution Earth observation satellites [5,6,7]. The GF-1 wide field view (WFV) cameras can acquire data that are highly valuable for drought dynamic monitoring because of their high spatial resolution, wide coverage and high revisit frequency (Table 1). However, the application of GF-1 data to agricultural remote sensing monitoring is still at an initial stage, and almost no literature exists to date on estimating drought from the GF-1 WFV data.

Many estimation algorithms by satellite data have been developed, but for satellites with four visible and near infrared bands (such as GF-1), a simple and effective method should be mentioned, which is two dimensional spectral space based on reflectance of red and near-infrared wavelengths. In as early as the 1970s, researchers had begun to study the patterns of changes in vegetation indices in the red- near-infrared two-dimensional (2D) spectral space [8]. Taking advantage of the reflective and absorptive features of the canopy and bare soils, Ghulam et al. [9] proposed a simple and effective perpendicular drought index (PDI), which is based on the red and near infrared bands. Qin et al. [10] estimated the PDI from Moderate Resolution Imaging Spectroradiometer (MODIS) data and used it to monitor the drought conditions in North China. Combining several characteristic parameters of the areas (e.g., soil and hydrological parameters), these authors quantitatively classified the PDI into several drought levels and found that the drought distribution derived from the PDI was consistent with the actual conditions. Zhu et al. [11] used 250-m-resolution data acquired by the Medium Resolution Spectral Imager (MERSI) sensor aboard the new-generation FY-3A satellite, which was developed independently in China, and the PDI to monitor the drought conditions in the central and eastern Inner Mongolia Autonomous Region during the summer of 2009. Similarly, these authors found a relatively high correlation between the PDI and the soil moisture content (SMC) at a depth of 20 cm. The study conducted by Chen et al. [12] in 2009 showed that the PDI derived from HJ/charge-couple device (CCD) data could be used to achieve effective monitoring of drought conditions, which demonstrates the effectiveness of HJ satellite data in disaster monitoring. Based on monitoring data acquired by the MODIS/Terra, FY-3B/MERSI and HJ/CCD sensors as well as the corresponding measured SMC data, Jiang et al. [13] used the PDI to comparatively analyze the sensitivity and reliability of these sensors in monitoring the temporal and spatial distributions of droughts and found that the PDI was most highly correlated with the relative soilmoistureinthe0–20 cm soil layer.

However, these authors also found that the PDI was less effective at monitoring the drought conditions in surface cover types that vary from bare soil to densely vegetated agricultural fields and are characterized by non-flat topography with different soil types. By considering the effects of vegetation cover, Ghulam et al. [14] proposed a modified PDI (MPDI) based on the red–near-infrared spectral space of Enhanced Thematic Mapper Plus (ETM+) data, through validation with data from Inner Mongolia and Ningxia, found that the MPDI was relatively highly accurate. Feng et al. [15] derived the PDI and MPDI from 30-m-resolution HJ-1A/1BCCD data and found that compared with the PDI, the MPDI was more sensitive to drought changes and more effective at monitoring the drought conditions in areas with relatively high fractional vegetation coverage (FVC) than the PDI. Moreover, they noted that the shape and characteristics of the soil line were related to the soil type, fertilization conditions, climatic characteristics and vegetation type. Shahabfar et al. [16,17] found high correlations of the MODIS-derived PDI and MPDI with the meteorological drought index, the vegetation condition index and the crop water index for Iran, which is an arid–semiarid country in Asia. Zhang et al. [18] validated the MODIS-derived MPDI for North China and found that the MPDI was highly effective at reflecting the drought conditions and was most highly correlated with the SMC at a depth of 10 cm. Li and Tan [19] validated the MPDI, which was composed of the perpendicular vegetation index (PVI) and the PDI derived from ETM+ data measured in Hubei Province, China, finding that the MPDI was more effective in high-FVC areas. Large amounts of usable high-resolution data are becoming available in China and elsewhere, which are often collected in only four bands, three in the visible spectrum and the near-infrared band. Therefore, the bright application prospects are bright for drought monitoring methods based on visible- near-infrared spectral space information.

In recent years, China has successively launched the new-generation GF series of Earth observation satellites, collecting data in the blue, green and red bands of the visible spectrum as well as the near-infrared band; these satellites have notably improved not only the spatial and temporal scales but also their range and accuracy of earth observations. Furthermore, these satellites have advanced the application and service capability of remote sensing in disaster reduction, but there is currently no drought monitoring model is currently based on GF-1 data. In this study, to improve their application potential in drought monitoring, the shortcomings and issues of the normalized difference vegetation index (NDVI)-based MPDI are considered. Soil texture distribution data and new vegetation indices are first introduced to not only reduce the effects of soil background and the saturation problem of NDVI in high-FVC areas but also increase the stability and comparability between areas. The study estimates a two-band enhanced vegetation index (EVI2)-based MPDI is established that can make full use of the advantages of GF-1 data and demonstrate the effectiveness of GF-1 WFV data in drought monitoring at fine temporal and spatial scales.

## 2. Study Area and Data

### 2.1. Study Region

**Figure 1:**
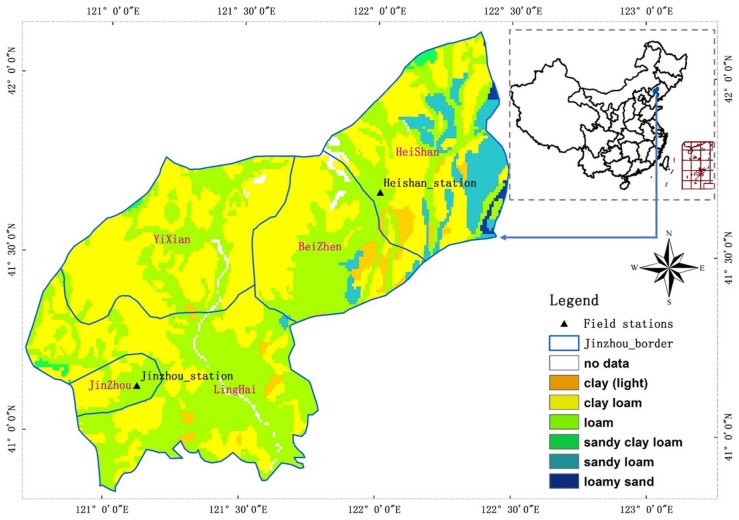
Study area and field stations distribution.

The Jinzhou region (including Yi, Heishan, Beizhen, Linghai and Jinzhou City) in Liaoning Province was selected as the study area (Figure 1). Topographically, it is high toward the northwest and low toward the southeast with the terrain sloping from northwest to southeast and consisting of a low-mountain, a hilly and a plains section. Situated in eastern Eurasia, Jinzhou has a warm temperate-zone semi-humid climate with predominant westerlies and subtropical atmospheric circulation systems. As a continental monsoon area, Jinzhou has four distinct seasons that are characterized by warm and windy springs, hot and rainy summers, warm-cool and sunny falls and cold and dry winters as well as concentrated precipitation and a clear monsoon season. Jinzhou has an annual mean temperature of 7.8–9.0 °C, which decreases from south to east, an annual maximum temperature of 41.8 °C, an annual minimum temperature of −31.3 °C, a 144–180-day frost-free season and an annual mean precipitation of 567 mm (the precipitation is uneven throughout the four seasons, with 60–70% occurring in the summer).

### 2.2. Data and Preprocessing

#### 2.2.1. Satellite Data

GF-1, which is the first satellite in the high-resolution Earth observation system, a major science and technology project of China, was successfully launched from the Gansu Jiuquan Satellite Launch Center at 12:13 on 26 April 2013. The GF-1 satellite uses the CAST2000 compact satellite platform technology and is equipped with two 8-m-resolution (2-m panchromatic resolution) multispectral high-resolution cameras and four 16-m-resolution multispectral WFV cameras (four sensors: WFV1, WFV2, WFV3 and WFV4). The GF-1 satellite orbits at an altitude of 645 km. A 25° swing of a GF-1 camera corresponds to a visual range of 700 km. The GF-1 satellite has a revisit period of four days. The WFV cameras can cover the entire globe once every four days without requiring the satellite to swing. The greatest advantage of the GF-1 satellite is its combination of a short revisit period, high resolution and wide field of view (Table 1) [5].

GF-1 data can be queried and downloaded through the Land Observation Satellite Data Service Platform of the China Center for Resource Satellite Data and Applications (CRESDA) [20]. In this study, GF-1/WFV imagery of 31 scenes of the study area from May to September 2013 and 2014 with less than 5% cloud cover were collected and downloaded (Table 2). The image preprocessing mainly included radiometric calibration, fine geometric correction and atmospheric correction. In the radiometric calibration process, based on the calibration coefficients provided by CRESDA, the digital value of channel observations was converted to apparent radiance data or reflectance to eliminate or reduce the difference between the sensor measurements and spectral radiance. The corrected ZY-3 data were used as reference imagery to perform the fine correction to reach a planar accuracy of less than one pixel in plain, which meets the sub-pixel accuracy requirements for multi-temporal remote sensing image classification. Based on the spectral response functions and half-wavelengths for the bands of the GF-1 satellite payloads, the Fast Line-of-sight Atmospheric Analysis of Spectral Hypercube (FLAASH) module in the ENVI5.1 software [21] was used to perform the atmospheric correction to eliminate the effects of the atmosphere, illumination and scattering by aerosols on the reflections by ground objects.

Despite efforts to select cloudless imagery, some areas in the selected imagery were still cloud covered. Based on an analysis of the spectral characteristics of the GF-1 data in each band, a simple multi-threshold discrimination method was employed to detect clouds and remove cloud-polluted pixels. The pixels with reflectance greater than 0.25 in the red band and greater than 0.45 in the near-infrared band and an NDVI less than 1 were identified as cloud pixels and subsequently removed. In addition, a buffer zone method was employed to eliminate cloud-polluted pixels, and five-pixel buffer zones were established to filter the pixels near the cloud pollution.

#### 2.2.2. Field Data

The relative soil water content (RSMC) data acquired at ground observation stations were used to validate the drought indices (Dis) established based on various vegetation indices and select the optimal drought model. RSMC is the ratio of soil water content to field water capacity. Ten-day RSMC (at a depth of 0–20 cm) data acquired at two observation stations, the Heishan station and the Jinzhou station, from May to September 2013 were downloaded from the data sharing website of the China Meteorological Data Service Center [22]. Each data point was examined, and invalid data points (marked with −9999) were removed. Based on the longitude and latitude of each station, the monitoring results for the corresponding location obtained using each drought model were extracted from the raster remote sensing drought monitoring data.

The conventional field drought assessment data were referred to the drought-affected region data. The field-based drought-affected crop areas in the study region were published in early August 2014, and downloaded from the website of the Liaoning Provincial Department of Water Resources (LNDWR) [23]. The drought-affected field areas were collected and submitted from various villages to counties and cities, which requires considerable manpower and material resources. These data, which are field-derived, regional, manual, and experiential observations based on crop drought status, and provide a suitable and exact description of drought severity. The different degree of drought-affected crops area in the Jinzhou region and five counties were included. Here we recommended these reference data for evaluating the GF-1 WFV-based drought model results on a county scale. 

#### 2.2.3. Auxiliary Data

Soil texture was obtained from the Harmonized World Soil Database (HWSD) [24], which is a 30 arc-second raster database with over 15,000 different soil mapping units. This database combines existing regional and national updates of soil information worldwide with the information contained within the 1:5,000,000 scale FAO-UNESCO Soil Map of the World (FAO, 1971–1981). This was the result of collaboration by the FAO with the International Institute for Applied Systems Analysis (IIASA); ISRIC-World Soil Information; Institute of Soil Science; Chinese Academy of Sciences (ISSCAS); and the Joint Research Centre of the European Commission (JRC). In present paper, the USDA soil type data were extracted and resampling into high resolution for building the soil lines in different soil types. The original soil type data were defined by 13 classes: C1-clay (heavy), C2-silty clay, C3-clay (light), C4-silty clay loam, C5-clay loam, C6-silt, C7-silt loam, C8-sandy clay, C9-loam, C10-sandy clay loam, C11-sandy loam, C12-loamy sand and C13-sand.

The precipitation data used in this research is the Climate Hazards Group Infrared Precipitation with Stations dataset (CHIRPS) [25,26], which is a fine-resolution (approximately 5 km × 5 km) and temporal range from the first pentad of January 1981 through the near-present. This product is a combination of Climate Hazards Group precipitation climatology [27], satellite infrared measurements, and rain gauge measurements. It has been validated and used in many researches [28,29,30], and is recommended as reference data in this study.

The crop distribution map for the study area can be obtained from the CropWatch bulletin [31], which is published quarterly [31,32]. This bulletin reports many climatic and remotely sensed indicators produced from the CropWatch system and their use in the analysis of climatic and crops condition assessments, providing accurate and timely information essential to food producers, traders and consumers [31,32]. These indicators include maps of cropped and uncropped arable land maps for different periods is one of those indicators that can be directly utilized. The maps comprise raster data, with a value of 1 and 0 indicating cropped area and unplanted areas, respectively. The crop distribution data for the Jinzhou area is extracted for use in masking the non-crop area.

## 3. Methodology

### 3.1. Soil Lines Determination

Because the characteristics of a soil line are related to the soil optical properties, the slope of a soil line may be vary with the soil type; consequently, there are differences in the drought results. It is generally assumed that a soil line is a straight line (the expression of the relationship between the reflectance in the near infrared and red bands) [14,15,16,17]. These researchers found that the soil line can be approximately expressed by Equation (1), as follows:

(1) $$R_{NIR}=M\times R_{Red}+I$$

where, *M* is the slope of the soil line, I is the interception on the vertical axis and $R_{Red}$ and $R_{NIR}$ are the reflectance of the red and near-infrared channels, respectively.

The distribution of soil line is highly dependent on the soil type, soil texture distribution data from HWSD database (detailed information seen in Section 2.2.3) was firstly employed for reducing error by different soil types. These data refer to six soil types, C3, C5, C9, C10, C11 and C12, with an area percentage of 3.2, 44.1, 44.2, 0.5, 7.5 and 0.5 of the entire study region, respectively. Because more sampling points were needed to fit the soil lines and relatively insignificant differences occurred among the soil types in this study region, soil types C3 and C5 were combined to form soil class CI, and soil types C9, C10, C11 and C12 were combined to form soil class CII. Soil lines were then established for CI and CII. Then new slope and interception of soil line for two classes (CI and CII) were generated by multi-temporal GF-1 data from the red and near-infrared bands, masked by the crop planting area, which is required and suitable for MDPI based on GF-WFV data. 

### 3.2. Candidate Vegetation Indices

In the present study, the eight most extensively used vegetation indices were collected, organized (Table 3), and used to establish drought models based on the different vegetation indices. Through comparison and an adaptability analysis, vegetation index with the relatively highest sensitivity to the MPDI was selected to modify the vegetation effects on the PDI, and a new MPDI based on the optimal vegetation index is established to improve the model performance of crop drought monitoring.

### 3.3. GF-1 WFV-Based MPDIs 

The scatter plot of the atmospherically corrected near-infrared and red reflectance spectrum demonstrated a typical triangle shape [9,10,14,17], which caused the construction of a vegetation coverage and drought monitoring index based on red and near-infrared spectral reflectance space. Ghulam et al. [9,10] was the first to design a PDI based on a red- and near-infrared spectral space for drought monitoring. Significant relationships exist between these indices and the soil moisture over different study areas that differed with vegetation coverage that varied from full vegetation cover to bare soil. These authors recommended applying these methods as drought monitoring indices in arid or semi-arid areas. The PDI can be used to effectively monitor surface drought conditions. It is calculated using the following mathematical equation:

(2) $$PDI=\frac{1}{\sqrt{M^{2}+1}}\left(R_{Red}+M\times R_{NIR}\right)$$

where *M* is the slope of the soil line, I is the interception on the vertical axis and $R_{Red}$ and $R_{NIR}$ are the reflectance of the red and near-infrared channels, respectively.

Vegetation has a high capacity to scatter light at many times in the visible and near-infrared bands. The PDI cannot effectively depict the drought conditions in vegetation area; and there are some limitations that challenge the performance of the PDI in areas whose surface cover types vary from bare soil to densely vegetated agricultural fields. Ghulam et al. used the FVC to express the vegetation factor and established an MPDI model [14]. A similar approach was adopted in our paper. Several studies found that strong correlations occur between FVC and the scaled NDVI [40]; later Jiang et al. [41] indicated that the scaled NDVI method would overestimate FVC in most cases by analyzing the spatial scale dependencies of the NDVI and the various forms of the relationships between the vegetation indices and FVC. We used the power function of the scaled NDVI to obtain FVC, as proposed by Baret et al. [42], and then acquired the several FVCs that could be calculated via the use of various candidate vegetation indices. The MPDI can be calculated by different candidate FVCs and vegetation indices, as given in the following equations:

(3) $$MPDI=\frac{R_{Red}+M\times R_{NIR}-FVC\times \left(R_{v,\;Red}+M\times R_{v,NIR}\right)}{\left(1-FVC\right)\sqrt{M^{2}+1}}$$

(4) $$FVC=1-{\left(\frac{VI_{max}-VI}{VI_{max}-VI_{min}}\right)}^{\theta }$$

where FVC is the fractional vegetation cover, VI is the vegetation index, $VI_{max}$ and $VI_{min}$ correspond to vegetation index value of bare soil and full vegetation coverage, repspectively; M is the slope of the soil line obtained from a linear regression of the soil points based on the red- near-infrared spectral space; and $R_{v,\;Red}$ and $R_{v,NIR}$ are pure vegetation reflectance in the red and near-infrared bands, respectively. For known vegetation growth, $R_{v,\;Red}$ and $R_{v,NIR}$ are determined as 0.05 and 0.5 by Ghulam’s result [10]; and θ is the regulatory factor for the vegetation index, which is set to 0.6175 from Ghulam et al. [10]. The MPDI values range from 0 to 1. The greater the PDI is greater with increased drought severity, and vice versa.

Initially, for the MPDI, the NDVI-based FVC is used to correct the effects of vegetation on the drought model’s monitoring results [40]. However, the NDVI is unfavorable for high-FVC areas due to a severe red light saturation problem, which results in an overestimation of the NDVI in low-FVC areas and an underestimation of the NDVI in high-FVC areas [43,44,45]. So besides of NDVI, other seven candidate vegetation indices (introduced in Section 3.1) were also considered here to optimize the best one and construct a corresponding MPDI model to improve its drought monitoring performance for FVC areas.

## 4. Results

The development of the crop drought monitoring algorithm for the GF-1 WFV data consisted of new soil lines for two soil classes in the study area, with their average slopes used to build the several MPDI models based on the various vegetation indices. Multiple MPDI models were established based on multiple vegetation indices by analyzing multiply vegetation indices responses to drought results. The optimization and presentation of the EVI2-based MPDI to estimate the drought extent from GF-1 WFV data were finished by RSMC and CHIRPS precipitation data. Finally, the EVI2-based MPDI for the GF-1 WFV data was validated with ground measurements.

### 4.1. Quantitative Analysis of the Soil Lines

**Figure 2:**
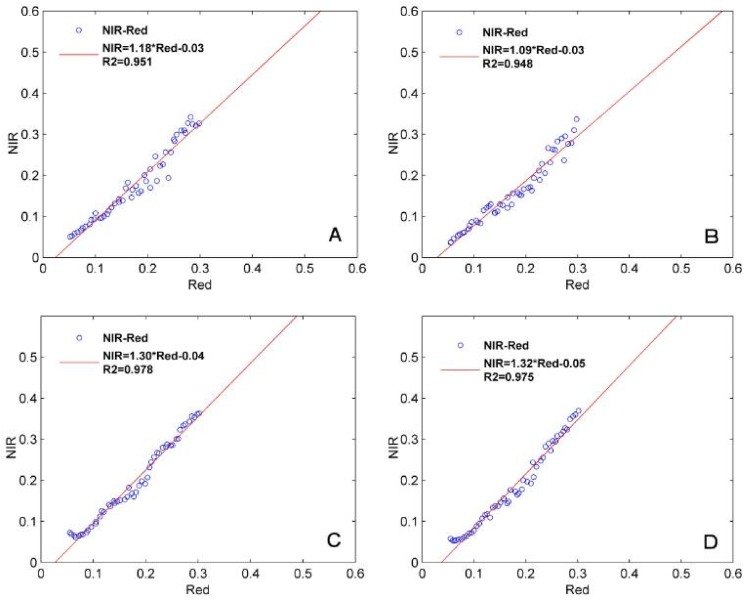
Fitting of soil lines in two types and two dates, (**A**) CI class in 9 August 2013; (**B**) CII class in 9 August 2013; (**C**) CI class in 2 August 2014; (**D**) CII class in 2 August 2014.

In this study, the soil lines were established for two classes with GF-1 data from 2013 to 2014. Figure 2 shows the relation of the red and near infrared band data and the fitting of the two soil lines for 9 August 2013 and 2 August 2014 in the study area. The correlation of determination of class CI is up to 0.951 and 0.978 in two dates, and class CII is 0.948 and 0.975. We can obtain the following soil line slopes from the fitting results: 1.09 and 1.18 (CI and CII) for 9 August 2013, and is 1.30 and 1.32 (CI and CII) for 2 August 2014.

**Figure 3:**
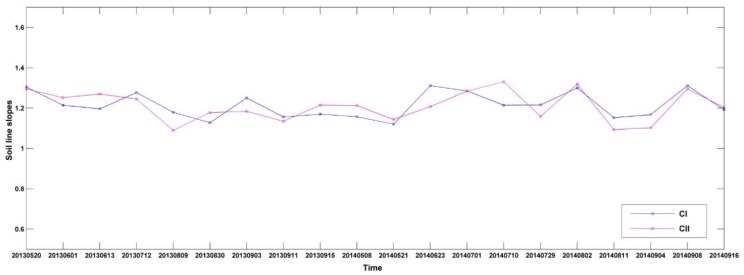
Soil line slopes for soil type of CI, CII.

The soil lines slopes changes and their distributions at different times can be found in Figure 3. The mean and variance of the slope are also calculated for each soil line. The slope of the soil line established for soil classes CI and CII averages 1.22 and 1.21, respectively, with a variance of 0.005 and 0.006 and no significant difference between the mean or variance of the two class of results. Furthermore, the slopes for the crop growing season show little difference within different time periods from 2013 to 2014. Therefore, the soil line slopes in CI and CII are relatively stable over the study area.

### 4.2. Preferred MPDI Model

RSMC can characterize whether a crop has suffered from water stress, which is closely related to the drought conditions. Lower RSMCs indicate that crop have suffered more severe drought, and vice versa. In this study, 10-day RSMC point-data were acquired at two agro-meteorological observation stations from May to September in 2013. It was employed to sensitivity analysis of variable vegetation indices to drought results by the correlation with the MPDI model based variable vegetation indices, then the optimal one was found to estimate the drought model.

The eight previously established VI-based drought monitoring models were compared to the RSMC data measured at the two ground stations in 2013 and 2014. The validation results are as follows. The Person correlation coefficients (Rs) between different MPDI models (based on GEMI, MSAVI, NDVI, PVI, SAVI, TSAVI, EVI and EVI2) and the RSMC measured at the Heishan station are −0.77, −0.79, −0.78, −0.77, −0.78, −0.79, −0.85 and −0.87, respectively. The R values between these MPDI models and the RSMC measured at the Jinzhou station are 0.79, −0.73, −0.80, −0.81, −0.81, −0.79, −0.86 and −0.89, respectively.

**Figure 4:**
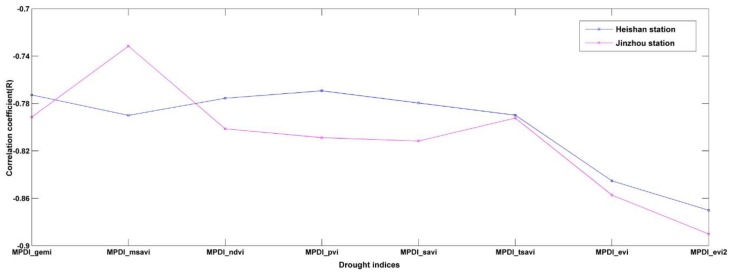
Correlation coefficients between different drought indices with RSMCs measured at the Jinzhou and Heishan meteorological stations.

**Figure 5:**
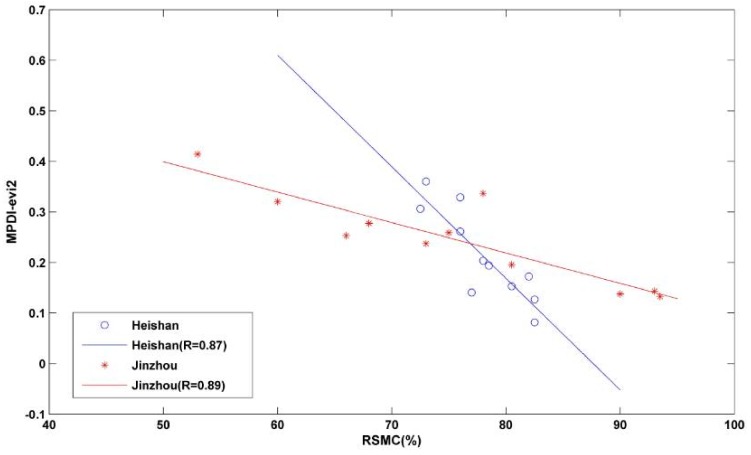
Scatter plot of MPDI_evi2 with RSMCs measured at the Heishan and Jinzhou agro-meteorological stations.

As shown in Figure 4, compared to the other vegetation indices, EVI and EVI2 are more highly correlated with RSMC, with the correlation between the EVI2 and the RSM being the highest. The R values between the EVI2 and the RSMC measured at the two stations are 0.87 and 0.89, respectively, as shown in Figure 5. The EVI2 was developed based on EVI. EVI equation is composed of the blue, red and near-infrared bands, whereas EVI2 is a development based on EVI that uses the red and near-infrared bands to represent the blue band in EVI. We discovered that the EVI2-based MPDI is more sensitive to the RSMC, and is therefore appropriate for refined drought monitoring. 

In addition, the drought may be caused from precipitation deficits, so which can reflect crop drought suffered status to some extent. Here, wetter year of 2013 and drier year of 2014 were selected for analyzing the response of several MPDI models to precipitation; and results from linear regression analysis indicated that all MPDI models based variable vegetation indices have a statistically significant relationship with precipitation data in general. Correlation coefficients between different drought indices with precipitation from CHIRPS (detailed introduction in Section 2.2.3) at the Jinzhou and Heishan meteorological stations can be found in Table 4. 

**Figure 6:**
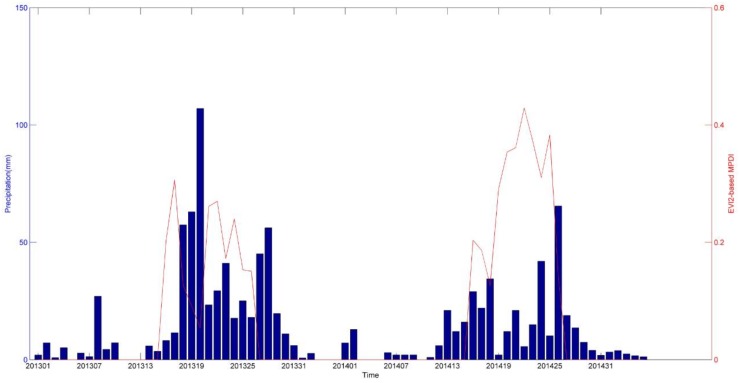
Temporal distribution of EVI2-based MPDI and precipitation during 2013 to 2014 at the Heishan meteorological station.

**Figure 7:**
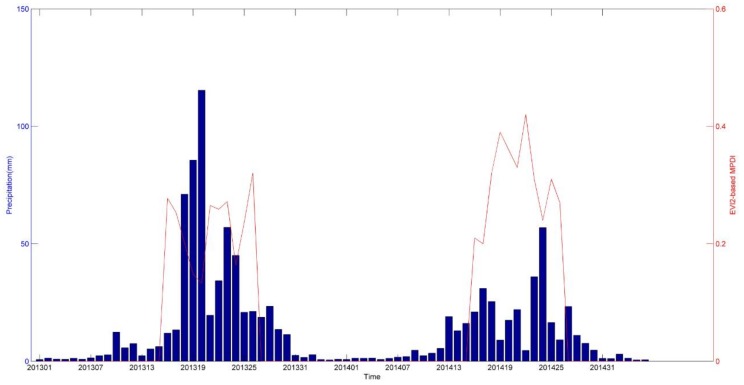
Temporal distribution of EVI2-based MPDI and precipitation during 2013 to 2014 at the Jinzhou meteorological station.

EVI2-based MPDI has the strongest correlation with precipitation in Heishan and Jinzhou station, and which of coefficient is little higher than EVI-based MPDI and precipitation. As to EVI2-based MPDI, We showed EVI2-based MPDI values (from May to September) and related precipitation (from January to December) in two locations in Figure 6 and Figure 7. The EVI2-based MPDI demonstrated a similar temporal trend in general, i.e., a higher index values followed less rainfall and lower values after major precipitation events. In other words, during 2013 to 2014, a drop in the MPDI corresponded to a significant rainfall occurred in previous days, or vice versa. There have significant correlation of changes of EVI2-based MPDI value and corresponding rainfall. Briefly, EVI2-based MPDI values in 2013 are mostly low than results of 2014, it means that heavier drought happened in 2014.

### 4.3. Results of EVI2-Based MPDI

The Jinzhou area is a drought-prone city, where a severe drought occurred in 2014 [26,46]. In this study, the crop growing period of 2014 is selected to display the trends of the temporal and spatial changes in the drought conditions and exploit the application potential of the GF-1 data. The slope of soil lines is from Section 4.1, the drought results can be obtained from the EVI2-based MPDI originated from Equations (3) and (4). 

Previous publications [9,10,47] classified the MPDI values into three drought categories, which are severe drought, mild drought and normal (the thresholds for the drought severity classifications can be found in Table 4). The field-based drought-affected crop data have four classifications (with an added moderate drought class); thus we made little change regarding the reference categories considered for comparability with the field-based drought-affected crop data, and defined the moderate and mild drought class are 0.35–0.4 and 0.3–0.35, respectively. The threshold used to classify drought severity for the EVI2-based MPDI from the GF-1 WFV data is showed in Table 5. The temporal and spatial drought distributions in 2014 are shown in Figure 6.

**Figure 8:**
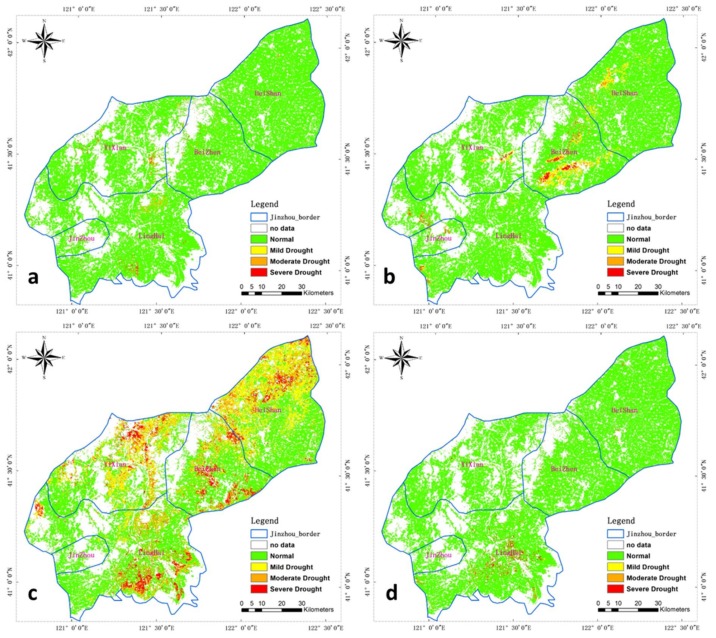
Spatial distribution of drought for EV2-based MPDI in different 2014 crop growing stages: (**a**) 6 June, (**b**) 1 July, (**c**) 2 August and (**d**) 9 September.

During the early stage of crop growth, drought occurred in few areas, those areas are east and west of Yi county, as well as north and south of Linghai City as shown in Figure 8a. In early July, crop planting area suffered different degrees of drought in Beizhen city, and slight drought in northwest Heishan, south Yi and west of Linghai (Figure 8b). Later, critical crop growing stage of July to August, the drought extent and severity are largely increasing, we know that severe drought had occurs through the entire Jinzhou area, except for parts of areas in south Heishan, Beizhen, south Yi, west Linghai and Jinzhou City (Figure 8c). This pattern is consistent with the drought characteristics of local governmental department [22]. From Figure 8d, the drought is obviously decreasing in most areas, which are in the late stage of crop growing.

### 4.4. Validation of EVI2-Based MPDI

The accuracy of the drought results based on the EVI2-based MPDI for the GF-1 data was evaluated using the drought statistical results of the local governmental department. These data were from a drought-affected crop area, with varying degrees of drought in sub-areas. Bias can be calculated for the drought-affected crop area with the data from the local governmental department and the EVI2-based MPDI model; and the percentage of bias toward the drought-affected crop area by a local statistics department is used. Comparisons of the proposed model and field observation data are made for bias and the percentage of bias. Table 6 summarizes the drought-affected crop area by the use of the LNDWR and the EVI2-based MPDI model, as well as comparisons in five sub-areas and for different drought degrees affecting the entire region.

Small differences occur between the drought-affected crop area from LNDWR and that from the proposed model results for the five sub-areas (−3.4, 1.9, 4.4, 1.8 and 1.5 thousand ha, Table 3). The percentage of bias for the entire region is approximately 4.8%, and the highest values is −21.8% in Jinzhou City. In addition, slightly higher differences exist between the statistical data and remote sensing monitoring results with respect to minor and severe drought conditions (27.1% and −24.7%). Generally, for the whole study region, the percentage of the bias toward the field drought-affected crop area is 4.6% (at an accuracy above 95%), which is good performance.

## 5. Discussion

This paper modified the MPDI model for suitable refined crop drought monitoring from GF-1 data, and proposed an EVI2-based MPDI model. Different soil lines were simulated in varying soil types, and EVI2 outperformed the other vegetation indices in building the drought monitoring model. Evaluated by the field drought statistical data published by LNDWR, the proposed model for the GF-1 WFV data was shown to perform well for refined crop drought monitoring.

Our analysis demonstrated that small slope changes occurred with soil type; thus soil type impacts the drought results. However, this finding is only based on the conditions of the study area, in which the soil types vary relatively insignificantly. We should focus on large areas for confirming this conclusion. Former researchers believe that the differences may result from variation in the soil color, soil brightness and fertilization conditions [9]. In Section 4.1, we find that small changes in the slope of the soil line occur during several crop growing stage, which can be explained by different fertilization or weather conditions on d days. Questions remain regarding the influence of those small slope changes on the drought predictions, and the size of contribution to errors in the drought model. We should investigate these questions in the future.

This study found EVI2-based MPDI to be more sensitive to drought conditions in high-FVC areas than the NDVI-based MPDI. Because EVI2 is a modification of EVI, both the EVI and EVI2 are resistant to atmospheric interference and soil background noise, and comparatively little affected by the aerosol optical depth error [19,39]. In contrast, the used NDVI is prone to saturation in high-FVC areas, is relatively significantly affected by soil background in the low-FVC areas, and is unable to thoroughly remove atmospheric attenuation. Why does EVI perform slightly better than EVI2 in our study? Possibly, the blue band is more susceptible to atmospheric correction, and does not provide additional biophysical information on vegetation properties [39,48]. For this study we used the ENVI software to conduct, not build the specific atmospheric correction algorithm for the GF-1 WFV data; this algorithm would reduce the EVI performance twofold.

The EVI2 development was undertaken to decompose the original EVI equation by eliminating the blue band and assuming that the blue reflectance can be expressed as a function of the red reflectance [39]. The parameter values of the EVI2 equation are dependent on the average ratio of the red to blue band reflectance for MODIS data. Therefore, this ratio may be variable for other sensors with different spectral functions, and the results from GF-1 data may vary slightly by directly using EVI2 based on MODIS data. 

FVC is another parameter in the MPDI equation, besides the soil line slope. In this paper and previous studies, supposing that the FVC algorithm is powerful and can exactly express the vegetation cover condition and changes in a certain area, FVC is proxy of vegetation coverage influence on drought model and the linear mixture were widely used FVC estimation methods [44,45,48,49,50], but the question arises whether FVC adequately describes the vegetation influences on the drought; these models required a definition of the NDVI values of highly dense vegetation and bare soil, generally empirical thresholds were used, which may cause some uncertainty and difference in extreme cases. 

Other possible causes for the differences between the monitoring model results and the ground measurements existed. For example, the accuracy assessment of the drought monitoring model is an important issue, but actual drought values are presently difficult to obtain from field surveys. Therefore, we recommended point-based RSMC data and field drought-affected crop area data to evaluate the proposed model’s performance. Some errors maybe arisen. The scale for the point-based RSMC data at the ground stations is inconsistent between the footprint of remote sensing data and the point-based nature of the RSMC measured parameter. Furthermore, different calculation methods exists between the drought-affected crop area data from LNDWR and from the proposed model. A direct comparison of these data may result in errors. Another possible cause for the differences could be temporal discrepancies between the drought monitoring results and the field observation data. However, the former (drought-affected crop area from the proposed model) was calculated based on satellite data from 2 August 2014, whereas the latter (drought-affected crop area data from LNDWR) was for 5 August 2014, with little difference between the data collection dates.

## 6. Conclusions

By now almost no drought model based on GF-1 WVF data, so we illustrated the potential and perspective for GF-1 satellite. GF-1 satellite data have high-resolution with 16 m, the highest frequency is up to 4 days and wide field view, more available data in crop growing stage from same four sensors can be obtained, even we have more chance to capture high quantity data in rainy season. The drought distribution map for high-frequency and high-resolution is necessary, especially for precise agriculture. 

A refined crop drought monitoring model was developed based on WVF GF-1 data and validated over the Jinzhou area of Liaoning Province, China. Different soil line slopes were simulated, and we considered EVI2 the best suitable one. Different soil line slopes were reconstructed and applied to different soil types. The validation results show that the relative errors for the drought-affected crop areas calculated using the EVI2-based MPDI model and the field data from LNDWR are small for an entire large area (4.8%), and the bias percentage ranges from −21.8% to 14.7% in five sub-areas. The proposed model can be used to achieve effective refined crop drought monitoring within high-resolution and frequency over large areas, which can provide various types of reliable drought information (e.g., an accurate spatial distribution of drought-affected crops and the drought level in each pixel or region) to relevant drought management departments for drought prevention, reduction and relief.

## Figures and Tables

**Table 1:** Specifications of GF-1 WFV cameras.

Bands No.	Spectral Range	Spatial Resolution	Width	Revisit Period	Transit Time
1	0.45–0.52	16 m	800 km	4 days	10:30 a.m.
2	0.52–0.59				
3	0.63–0.69				
4	0.77–0.89				

**Table 2:** Used GF-1 WFV data list.

Transit Time	Scene No.	Data Information
2013/05/20	1	GF1_WFV1_E121.9_N41.3_20130520_L1A0000046801.hdf
2013/06/01	1	GF1_WFV1_E121.2_N41.3_20130601_L1A0000020121.hdf
2013/06/13	1	GF1_WFV1_E121.4_N41.3_20130613_L1A0000028258.hdf
2013/07/12	3	GF1_WFV3_E123.5_N42.2_20130712_L1A0000053576.hdf GF1_WFV2_E121.3_N42.6_20130712_L1A0000052402.hdf GF1_WFV2_E120.8_N41.0_20130712_L1A0000052403.hdf
2013/08/09	1	GF1_WFV1_E121.2_N41.4_20130809_L1A0000067755.hdf
2013/08/30	1	GF1_WFV4_E121.4_N41.8_20130830_L1A0000077556.hdf
2013/09/03	1	GF1_WFV4_E121.6_N41.8_20130903_L1A0000079519.hdf
2013/09/11	2	GF1_WFV3_E121.6_N42.2_20130911_L1A0000082577.hdf GF1_WFV3_E121.0_N40.5_20130911_L1A0000082578.hdf
2013/09/15	2	GF1_WFV4_E122.2_N41.8_20130915_L1A0000084339.hdf GF1_WFV4_E121.6_N40.1_20130915_L1A0000084340.hdf
2014/05/08	1	GF1_WFV1_E120.9_N41.3_20140508_L1A0000220193.hdf
2014/05/21	2	GF1_WFV3_E121.6_N42.2_20140521_L1A0000231625.hdf GF1_WFV3_E121.1_N40.6_20140521_L1A0000231626.hdf
2014/06/23	4	GF1_WFV4_E122.9_N41.8_20140623_L1A0000258242.hdf GF1_WFV4_E122.3_N40.1_20140623_L1A0000258243.hdf GF1_WFV3_E120.6_N42.2_20140623_L1A0000258228.hdf GF1_WFV3_E120.0_N40.6_20140623_L1A0000258229.hdf
2014/07/01	2	GF1_WFV3_E121.8_N42.2_20140701_L1A0000264733.hdf GF1_WFV3_E121.2_N40.6_20140701_L1A0000265941.hdf
2014/07/10	1	GF1_WFV4_E119.3_N41.8_20140710_L1A0000271938.hdf
2014/07/29	1	GF1_WFV1_E121.5_N41.3_20140729_L1A0000289542.hdf
2014/08/02	1	GF1_WFV1_E121.6_N41.3_20140802_L1A0000293356.hdf
2014/08/11	2	GF1_WFV3_E121.7_N42.2_20140811_L1A0000301287.hdf GF1_WFV3_E121.2_N40.6_20140811_L1A0000301288.hdf
2014/09/04	2	GF1_WFV2_E122.7_N41.0_20140904_L1A0000327811.hdf GF1_WFV1_E120.4_N41.3_20140904_L1A0000327798.hdf
2014/09/08	1	GF1_WFV1_E121.9_N41.3_20140908_L1A0000336275.hdf
2014/09/17	1	GF1_WFV3_E121.1_N42.2_20140917_L1A0000344707.hdf

**Table 3:** Candidate drought indices.

Name of Indices	Authors	Formula
Normalized difference vegetation index (NDVI)	Rouse et al., 1974 [33]	$\frac{R_{NIR}-R_{RED}}{R_{NIR}+R_{RED}}$
Perpendicular vegetation index (PVI)	Richardson & Wiegand, 1977 [2]	$\frac{R_{NIR}-\alpha R_{RED}-\alpha }{\sqrt{1+\alpha^{2}}},\;\alpha =1.253$
Soil adjusted vegetation index (SAVI)	Huete et al., 1988 [34]	$\frac{R_{NIR}-R_{RED}}{R_{NIR}+R_{RED}+L}\left(1+L\right),\;L=0.5$
Modified soil adjusted vegetation index (MSAVI)	Qi et al., 1994 [35]	$\frac{2R_{NIR}+1-\sqrt{1+{\left(2R_{NIR}+1\right)}^{2}-8\left(R_{NIR}-R_{RED}\right)}}{2}$
Transformed soil adjusted vegetation index (TSAVI)	Baret, F. and Guyot, G., 1991 [36]	$\alpha \left(R_{NIR}-\alpha R_{RED}-\beta \right)/{\left(\alpha R_{NIR}+R_{RED}-\alpha \beta +0.08\left(1+\alpha^{2}\right)\right)}_{}$
Global environment monitoring index (GEMI)	Pinty, B. and Verstraete, M., 1992 [37]	$\gamma \left(1-0.25\gamma \right)-\left(R_{RED}-0.125\right)/\left(1-R_{RED}\right)$, $\gamma =\left(2(R_{NIR}^{2}-R_{RED}^{2}\right)+1.5R_{NIR}+0.5R_{RED})/\left(R_{NIR}+R_{RED}+0.5\right)$
Enhanced vegetation index (EVI)	Liu, H.Q., Huete, A.R., 1995 [38]	$2.5\times \frac{R_{NIR}-R_{RED}}{R_{NIR}+6\times R_{RED}-7.5\times R_{BLUE}+1}$
Two-band enhanced vegetation index (EVI2)	Jiang et al., 2008 [39]	$2.5\times \frac{R_{NIR}-R_{RED}}{R_{NIR}+2.4\times R_{RED}+1}$

Note: $R_{RED}$, $R_{NIR}$ and $R_{BLUE}$ is atmospheric corrected reflectance of red, near-infrared and blue band, respectively.

**Table 4:** Correlation coefficients between different drought indices with precipitation from CHIRPS at the Jinzhou and Heishan meteorological stations.

Pearson’s Correlation Coefficient (R)	MPDI_gemi	MPDI_msavi	MPDI_ndvi	MPDI_pvi	MPDI_savi	MPDI_tsavi	MPDI_evi	MPDI_evi2
Heishan_station	−0.68	−0.67	−0.67	−0.61	−0.63	−0.64	−0.71	−0.73
Jinzhou_station	−0.66	−0.64	−0.66	−0.56	−0.53	−0.54	−0.70	−0.70

**Table 5:** Classification of the EVI2-based MPDI and the MPDI from the Ghulam and Qin Qiming drought indices.

Drought Class	MPDI from Former Publishes	EVI2-Based MPDI in This Paper
Severe drought	>0.4	>0.4
Moderate drought	-	0.35–0.4
Mild drought	0.3–0.4	0.3–0.35
Normal	0–0.3	0–0.3

**Table 6:** Drought-affected crop area by LNDWR and the EVI2-based MPDI model, as well as comparisons.

Comparison Items	Drought-Affected Crop Area from LNDWR (Thousand ha)	Drought-Affected Crop Area from MPDI_evi2 (Thousand ha)	Bias *	Percentage ** (%)
Jinzhou	15.5	12.1	−3.4	−21.8
Beizhen	16.7	18.6	1.9	11.6
Heishan	30.0	34.4	4.4	14.7
Yi	32.3	34.1	1.8	5.7
Linghai	43.3	44.8	1.5	3.5
Jinzhou region	137.7	147.0	9.3	4.6
Severe drought in the Jinzhou region	43.3	32.6	−10.7	−24.7
Moderate drought in the Jinzhou region	29.3	31.7	2.4	8.1
Slight drought in the Jinzhou region	65.1	82.7	17.6	27.1

Note: Bias * is the difference in the drought-affected crop area between LNDWR and the EVI2-based MPDI model, Percentage ** is the percentage of bias toward the drought-affected crop area from LNDWR.
